# Lack of uniform diagnostic criteria for cervical radiculopathy in conservative intervention studies: a systematic review

**DOI:** 10.1007/s00586-012-2297-9

**Published:** 2012-04-25

**Authors:** Erik J. Thoomes, Gwendolijne G. M. Scholten-Peeters, Alice J. de Boer, Remy A. Olsthoorn, Karin Verkerk, Christine Lin, Arianne P. Verhagen

**Affiliations:** 1 Research Group Diagnostics, University of Applied Sciences, Breda, The Netherlands; 2 Department of General Practice, Erasmus Medical Centre, Rotterdam, The Netherlands; 3 University of Applied Sciences, Rotterdam, The Netherlands; 4 The George Institute for Global Health, Sydney Medical School, The University of Sydney, Sydney, Australia

**Keywords:** Cervical radiculopathy, Diagnostic labelling, Review, Definition

## Abstract

**Purpose:**

Cervical radiculopathy (CR) is a common diagnosis. It is unclear if intervention studies use uniform definitions and criteria for patient selection. Our objective was to assess the uniformity of diagnostic criteria and definitions used in intervention studies to select patients with CR.

**Methods:**

We electronically searched the Cochrane Controlled Trials Register, MEDLINE, EMBASE and CINAHL. Studies were included when evaluating conservative interventions in randomised clinical trials (RCTs) in patients with CR. Selection criteria and definitions for patients with CR were extracted and evaluated on their uniformity.

**Results:**

Thirteen RCTs were included. Pain was used as an inclusion criterion in 11 studies. Inclusion based on the duration and location of pain varied between studies. Five studies used sensory symptoms in the arm as inclusion criterion. Four studies used cervical range of motion and motor disturbances as inclusion criteria, while reflex changes were used in two studies. Three studies included patients with a positive Spurling’s test and two studies used it within a cluster of provocation tests.

**Conclusions:**

Criteria used to select patients with CR vary widely between different intervention studies. Selection criteria and test methods used are poorly described. There is consensus on the presence of pain, but not on the exact location of pain.

## Introduction

Cervical radiculopathy (CR) is a widespread diagnosis. Typically, CR is associated with symptoms of neck, shoulder, and upper limb pain as well as upper limb paraesthesia and weakness, which are attributed to cervical nerve root irritation. The clinical diagnosis of CR relies mainly on the outcome of history taking and a physical examination in which diminished muscle tendon reflexes, sensory disturbances, or motor weakness with dermatomal/myotomal distribution can be found [1].

Epidemiological data on CR are sparse [1]. A population-based study indicated that CR had an annual incidence rate of 107.3 per 100,000 for men and 63.5 per 100,000 for women, while the age-specific annual incidence rate reached a peak of 202.9 for the age group 50–54 years [2]. Another study reported a prevalence of 3.5 per 1,000 people and a peak annual incidence of 2.1 case per 1,000 people, which increased to a peak at age 50–59 years [3].

The aetiology in 70–75 % of cases is a foraminal compression of the spinal nerve. This can be due to several factors, including reduction in disc height and anterior and posterior degenerative changes of the uncovertebral and zygapophyseal joints [4]. The most common level of nerve root compression is C7, followed by C6; compression of roots C5 and C8 are less frequent [2, 5]. A herniated disc in the cervical spine accounts for only 20–25 % of the cases of CR [2, 5, 6]. CR as a direct result of cervical trauma or metastases is infrequent [7]. Although CR is a common diagnosis, there is still no consensus on the definition [8]. The differential diagnosis of CR can be extensive and includes many musculoskeletal or neurological conditions that may mimic the signs and symptoms of CR [1].

It has been suggested that CR is a diagnosis based on clinical impression, advanced testing, electrophysiology tests, or a combination of these tests [1, 9, 10]. There are no generally accepted, well-defined clinical criteria for the diagnosis of CR [1, 2]. A clear definition of terms is required to establish definitive diagnostic criteria for evaluating the (cost)effectiveness of treatment of patients with CR [2, 4, 6, 11]. An unambiguous diagnostic classification of CR is necessary to be able to select a homogeneous patient population for daily practice and research. It will facilitate communication and help in identifying subgroups of patients differing from the overall population in prognosis or treatment benefit.

Therefore, this systematic review aims to assess the uniformity of diagnostic criteria and definitions used in intervention studies (with at least one conservative treatment group) to select patients with CR.

## Methods

### Search strategy and selection criteria

We used the search from that identified from the search of our review on the effectiveness of conservative interventions in CR [12]. The search strategy followed the recommendation by the Cochrane Handbook for Systematic Review of Interventions [13]. Electronic searches included Cochrane Controlled Trials Register, MEDLINE, EMBASE, and CINAHL from 1966 up to October 2010. Manual searches of review bibliographies and reference lists of primary studies were undertaken to obtain possible studies not captured by the electronic searches. Two librarians together with a review author (ET) performed the electronic search (“Appendix”). Studies were included that evaluated a conservative intervention in a randomised clinical trial (RCT) in patients with CR. Different from our previous review [12], this time the type of outcome measures or the type of comparison interventions used were not taken as an exclusion criterion.

### Data extraction

From each included study, the diagnostic criteria used to define the diagnosis of CR (not criteria related to the intervention) and the definitions for CR were extracted.

Three reviewer authors (ET, RO, AdB) performed the data extraction independently, using a pre-determined data extraction form (available from the authors).

### Analysis

The criteria for patient selection were qualitatively and quantitatively evaluated on uniformity. We divided selection criteria into clinical symptoms, clinical testing, diagnostic imaging, and exclusion criteria. Clinical symptoms were subdivided into pain and sensory symptoms. Clinical tests were subdivided into pain provocation tests, changes in range of motion, and neurological examination, e.g. motor disturbances and reflex changes. We aimed to identify either corresponding or contradictory diagnostic tests and features of CR. Items were considered to correspond if they described the same test, cluster of tests, or feature in labelling “cervical radiculopathy” (e.g. “Spurling’s compression test” or “combined lateral flexion and extension”) provoking neck and/or arm pain. Our conclusion of consensus between criteria or definitions across the studies was (arbitrary) set at 75 %: if more than 75 % of studies set a certain criterion. Criteria were considered to be contradictory when the item was a reason for inclusion in one article and a reason for exclusion in another.

## Results

### Selection of studies

**Fig. 1:**
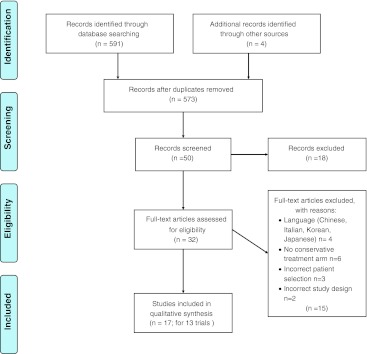
PRISMA flow diagram

Figure 1 (PRISMA Flow Diagram), [14] identifies the study selection process. We included 17 articles reporting on 13 studies [15–30]. Four articles reported on one single trial [23–26] and two articles were identical, but one was published in Dutch [31] and one in English [22].

Two studies included patients with no other specific selection criteria besides having CR [15, 29]. Table 1 presents the results of data extraction.

**Table 1:** Selection criteria of cervical radiculopathy

References	Study	Definition	Clinical symptoms	Clinical tests	Imaging tests	Exclusion
Atteya [15]	RCT, *n* = 20 I: EMG biofeedback traction C: conventional traction	NM	NM	Clinical examination and EMG studies	NM	NM
British Association of Physical Medicine [16]	RCT, *n* = 493; I1: cervical traction I2: positioning I3: collar C1: placebo tablets C2: shortwave diathermy (placebo heat)	NM	1. Pain in neck and arm (±paraesthesias) with (partial) root distribution and associated with limited and painful movements of the neck 2. Pain in the neck and arm of (partial) root distribution with paraesthesias, but without clinical evidence of abnormality in the neck	NM	NM	Symptoms due to local lesions; specific pathology, such as RA; abnormal neurological signs; injuries of the spine; use of steroids or phenylbutazone
Elnaggar et al. [17]	RCT, *n* = 30 I: intermittent traction + infrared therapy C: continuous traction + infrared therapy	A pathologic process, which has been defined as pain in the distribution of a specific cervical nerve root resulting from damage to either the dorsal or ventral nerve root or both	Unilateral radicular symptoms for at least 6 months and up to 2 years	NM	NM	NM
Fukusaki et al. [18]	RCT, *n* = 53 I: (consecutive) nerve blocks C: conventional therapy (oral medication and soft collar)	NM	Severe pain in the arm, neck, shoulder, and/or interscapular region	Restricted neck motion and positive results of the shoulder abduction test	Plain X-rays (revealing cervical degenerative changes including spur formation, hypermobility state, and/or disc narrowing). MRI revealing herniated intervertebral disc or disc bulging of either C3/4 or C6/7	Progressive neurological deficits, i.e. marked motor weakness, hyperaesthesia or abnormal tendon reflex. Myelopathic signs, i.e. muscle atrophy, pathological reflex, or gait disturbance
Jellad et al. [19]	RCT, *n* = 39; I1: manual traction + standard rehabilitation programme I2: mechanical traction + standard rehabilitation programme C: standard rehabilitation programme	Metameric neck pain, which radiates to the arms	NM	NM	CT and/or MRI and concordant radiographic results confirming herniated intervertebral disc and/or disc degeneration	History of surgery or bone–ligament damage to the cervical spine, shoulder disease (rotator cuff syndrome, capsulitis, acromioclavicular arthropathy, shoulder instability, or inflammatory arthritis) or carpal tunnel syndrome, ongoing or recent rehabilitation for the current CR, and the worsening of pain or intolerance in a manual cervical traction test performed
Joghataei et al. [20]	RCT, *n* = 30; I: electrotherapy, isometric exercises + traction C: electrotherapy, isometric exercises	A pathologic process, which has been defined as pain in the distribution of a specific cervical nerve root caused by nerve root compression	History of neck pain for more than 1 month Unilateral C7 radiculopathy C7 dermatomal numbness	The Spurling neck compression test accentuated the symptoms of C7 nerve root involvement	MRI: pathological lesions corresponding to the C7 nerve root	History of systemic disease such as RA, tuberculosis, cervical myelopathy, multiple sclerosis, stroke, ALS
Klaber Moffet and Hughes [21]	RCT, *n* = 100; I: traction; C: placebo traction	NM	Symptoms in the arm to be considered clinically indicative of a radiculopathy or brachialgia stemming from the neck	NM	NM	History of neck and arm pain <3 months; shoulder movement limited >25 % on the affected side; received any physiotherapy for the same problem in the last 6 months; previously had unsuccessful cervical traction; a systemic or other condition for which traction would normally be contraindicated
Kuijper et al. [22]	RCT, *n* = 205; I1: semi-hard collar I2: physiotherapy and home exercises C: no treatment	A common disorder characterised by neck pain radiating to the arm and fingers corresponding to the dermatome involved.	Symptoms for <1 month and arm pain on a visual analogue scale of 40 mm or more Radiation of arm pain distal to the elbow, and at least one of the following: Provocation of arm pain by neck movements; sensory changes in one or more adjacent dermatomes; muscle weakness in one or more adjacent myotomes; or diminished deep tendon reflexes in the affected arm	Neck movements	NM	Clinical signs of cord compression
Persson and Lilja [26]	RCT, *n* = 81 I1: surgical decompression + fusion C1: physiotherapy C2: cervical collar	NM	Cervicobrachial pain for more than 3 months Neurological examination to determine clinical level of radiculopathy	NM	X-ray and MRI or CT	Whiplash, traumatic injuries Serious diseases
Ragonese [27]	RCT, *n* = 30 I1: manual therapy (MT) I2: exercise I3: combination MT + Exc	Disorder of the cervical nerve root, most often the result of compression or inflammatory response from space occupying lesions, e.g. herniated disc or osteophyte	Chief complaint of neck and/or upper extremity symptoms; either distal or proximal to the elbow	Presence of four positive examination findings: positive Spurling test, positive distraction test, positive Upper Limb Tension Test for median nerve bias, and ipsilateral cervical rotation <60°	NM	Current medical condition such as current fracture, history of rheumatoid arthritis or osteoporosis, current bilateral upper extremity symptoms, evidence of central nervous system involvement, or history of cervical or thoracic surgery
Shakoor et al. [28]	RCT, *n* = 218 I: cervical traction, exercises, postural advice, collar, thiamine C: NSAIDs (naproxen), ranitidine, placebo traction, postural advice, collar, thiamine	NM	Pain in the neck and arm Symptoms having root distribution and associated with limited and painful movements of the neck	NM	NM	Symptoms due to local lesions; abnormal neurological signs; Specific pathology, such as RA; tuberculosis, injuries of the spine
Wong et al. [29]	RCT, *n* = 24 I: traction with closed loop EMG-feedback C: traditional traction with open loop EMG.	NM	NM	Undefined clinical testing and electrodiagnosis	NM	NM
Young et al. [30]	RCT, *n* = 81 I: manual therapy, exercise, and intermittent cervical traction. C: manual therapy, exercise, and sham intermittent cervical traction	Disorder commonly associated with cervical disc derangement or other space occupying lesion, resulting in nerve root inflammation, impingement or both	Unilateral upper extremity pain, paraesthesia, or numbness with or without neck pain	Three of four tests of CPR have to be positive: Spurling test; distraction test; Upper Limb Tension Test 1; ipsilateral cervical rotation <60°		History of previous cervical or thoracic spine surgery Bilateral upper extremity symptoms Signs or symptoms of upper motor neuron disease Medical “red flags” (e.g. tumour, fracture, rheumatoid arthritis, osteoporosis, prolonged steroid use) Cervical spine injections (steroidal) in the past 2 weeks Current use of steroidal medication prescribed for radiculopathy symptoms

*RCT* randomised clinical trial, *I* intervention, *C* control treatment, *CPR* clinical prediction rule, *VAS* visual analogue scale, *NM* not mentioned in the study

### Definitions

Six studies used a definition of CR [17, 19, 20, 22, 27, 30]. Two studies [17, 20] used identical definitions. Four definitions [17, 19, 20, 27, 30] mentioned nerve root compression resulting in neck pain radiating to the arm. We concluded that there was no consensus on a definition in the literature.

### Clinical symptoms

#### Pain

Eleven studies reported pain as selection criterion [16–23, 27, 28, 30]. One study reported that pain intensity should be above 40 mm on a 100-mm visual analogue scale (VAS) [22]. One study [28] mentioned using the inclusion criteria from another study [16].

The location of pain (arm and/or neck) was described in 11 studies as criterion [16–23, 27, 28, 30], but in two studies the location ‘cervicobrachial pain’ [23] and ‘neck pain’ [20] was not further explained. Two studies solely reported arm pain to be present [22, 30], of which one study stated that arm pain, with radiation distal to the elbow, plus at least provocation of arm pain by neck movements should be positive as selection criterion [22]. Six studies included patients with neck pain and arm pain [16, 18–20, 26, 28]. In three of these studies, patients also had to have nerve root distribution of the pain for inclusion [16, 27, 28].

Five studies described the duration of pain as selection criterion. These studies included patients with pain duration of <1 month [22], >1 month [20], <3 months [19, 21], or >3 months [23]. The total range of duration of pain ranged from 1 month to 1 year [15], from 6 months to 1 year [17], or from 1 month to 2 years [29].

We concluded that there was consensus (11 out of 13 studies; 85 %) on pain as a selection criterion, but no consensus on the exact location, intensity, or duration of pain. Only 6 out of 13 studies (46 %) require both neck and arm pain to be simultaneously present.

#### Sensory symptoms

There were five studies which used sensory symptoms as selection criteria [16, 20, 22, 23, 30]. One study mentioned using reflex disturbances, motor and sensory deficits, together with the distribution of pain, to determine the clinical level of radiculopathy [23].

Other inclusion criteria used were paraesthesia [16, 30], numbness [20, 30], and sensory changes [22]. A definition of these terms or what the symptoms include was often not explicitly outlined.

Three studies provided information concerning the location of the sensory symptoms. The C7 dermatome [20], one or more adjacent dermatomes [22], and symptoms in unilateral upper extremity [30] were mentioned. The exact location of the sensory symptoms was not further explained in any of the studies.

No study described whether the information on sensory symptoms was gathered during history taking or by physical examination. In conclusion, we found no consensus (5 out of 13; 38 %) across the studies about sensory symptoms as a selection criterion.

### Clinical tests

#### Pain provocation tests

Pain provocation tests were used in three studies to select patients with CR [20, 27, 30]. One study described a positive Spurling test as a sign indicating nerve root involvement [20]. Two studies used a clinical prediction rule for selection that included a cluster of four tests: Spurling test, distraction test, upper limb tension test, and ipsilateral cervical rotation <60°. For inclusion, three out of four provocation tests should be positive [27, 30]. Only one study described how the tests were performed and the criteria for positive testing [27].

We concluded that there was no consensus (3 out of 13 studies; 23 %) on pain provocation tests.

#### Range of motion

Four studies reported changes in range of motion of the neck as selection criteria [18, 27, 28, 30]. Two studies [27, 30] used the cutoff value of <60° of cervical rotation as proposed in a clinical prediction rule [9]. The reason for this cutoff point was not described. Other studies used ‘limited and painful movements of the neck’ or ‘restricted neck motion’ as selection criteria, but the way of testing, the kind of dysfunction, and cutoff points were unclear [18, 28]. We concluded that there was no consensus on this item.

#### Neurological tests, motor disturbances, and reflex changes

One study included patients who had motor disturbances or reflex changes [22], namely muscle weakness and ‘diminished deep tendon reflexes in the affected arm’. The assessment of muscle weakness was clearly described.

In conclusion, concerning clinical tests, we found no uniformity in studies in the criteria used to label patients as having CR.

### Diagnostic imaging tests

Imaging methods were used in four studies for the identification of patients with CR [18–20, 23].

All four studies used magnetic resonance imaging (MRI) in their selection criteria and two of these studies mentioned the use of MRI to reveal a herniated intervertebral disc [18, 19]. One study included patients with pathological lesions corresponding to the C7 nerve root detected by MRI, but the exact descriptions of the pathological lesions were unclear [20]. One study used MRI and radiographs (X-rays) as part of the neurological examination, but it was unclear when patients were regarded as eligible based on the imaging results [23].

In conclusion, imaging methods were not uniformly used in studies to label patients as having CR.

### Exclusion criteria

All but three studies explicitly stated exclusion criteria [15, 17, 29]. Eight studies excluded patients with specific pathology or medical ‘red flags’, although the examples mentioned differed [16, 18, 20, 21, 23, 27, 28, 30]. Planned imminent treatments (surgery or injection), previous injuries or surgery of the spine, use of medication, clinical signs of cord compression, abnormal neurological signs, or the inability to tolerate the planned intervention were also mentioned as exclusion criteria across the different studies.

Overall, many studies mentioned exclusion criteria, but we found no uniformity in the criteria used to exclude patients with symptoms of CR. No criteria were considered to be contradictory.

## Discussion

This systematic review found no uniformity in the definitions of CR. Six studies specifically defined CR. The most common definition mentioned nerve root compression resulting in neck pain radiating to the arm. We found consensus on one criterion for selecting patients with CR for RCTs using conservative therapy as an intervention: 11 out of 13 studies mentioned pain as a selection criterion. We found no consensus on the location of pain: 7 out of 13 studies mentioned a combination of neck and arm pain as a selection criterion, 2 studies mentioned neck and/or arm pain and 2 studies mentioned arm pain as a selection criterion. No criteria were considered to be contradictory.

### Comparison with the literature

In the literature, we found no other SR on selection criteria or definitions of CR. A qualitative review on the diagnosis of CR mentioned conventional neurologic examination findings (testing of strength, muscle stretch reflexes, and sensation) and cervical ROM as a part of the clinical examination procedure [9]. This review suggested the use of a test item cluster for diagnosing CR. A systematic review of the diagnostic accuracy of provocative tests of the neck for diagnosing CR suggested that, when consistent with the history and other physical findings, a positive Spurling‘s, traction/neck distraction and positive Valsalva’s test might be indicative of a CR, while a negative Upper Limb Tension Test might be used to rule it out [10].

Recently, a work group consensus statement from the North American Spine Society suggested defining CR from degenerative disorders as “pain in a radicular pattern in one or both upper extremities related to compression and/or irritation of one or more cervical nerve roots” [32]. This review found six studies mentioning definitions, all comprising a cluster of likely or possible symptoms including neck and arm pain and varying degrees of sensory, motor, and reflex changes. It is likely that the lack of a consensus definition for CR has contributed to the different findings with regards to the effectiveness of treatments for CR reported in the literature [12, 32, 33].

With regard to criteria for the inclusion of patients, many authors stress the value of elaborate history taking [9, 34]. Except on pain, however, we found no specific mentioning of history taking being included in the diagnostic criteria of studies.

Neck pain radiating into the arm or cervicobrachial pain is a common feature in CR, but not a distinguishable one per se, as it could well have other causes such as a thoracic outlet syndrome [35–37] or referred pain [37]. Nerve root compression is supposed to result in both neuropathic and nociceptive pain [38, 39]. Possibly, future selection criteria should also take these different natures of pain into account. A more specific definition of CR should therefore include an ability to distinguish the nerve root pain present in CR from other musculoskeletal disorders and other neurological conditions, such as brachial plexus pathology, pseudo-radicular pain or a peripheral nerve entrapment, or even non-specific neck pain [1, 6].

We did not find any mention of the use of validated questionnaires for distinguishing neuropathic from nociceptive pain such as the Neuropathic Pain Questionnaire (NPQ) [40], ID Pain [41], Leeds Assessment of Neuropathic Symptoms and Signs (LANSS) [42], Douleur Neuropathique 4 (DN-4) [43], or painDETECT [44]. Because neuropathic pain has a worse prognosis than nociceptive pain and there are treatments that specifically target neuropathic pain, future studies could consider using the questionnaires to select and tailor participants to different treatment strategies [45].

Diagnostic labelling could well include results from physical examination. Only five studies mentioned selection of participants based on clinical (provocation) tests [18, 20, 22, 27, 30]. However, there is a lack of primary studies investigating the accuracy of these tests. The reported heterogeneity between the various studies, as well as numerous methodological problems, precludes any strong recommendations for the use of these tests, especially in the primary care setting. In the absence of other clinical information or corroborating evidence, the value of these tests should therefore be interpreted with caution.

Diagnostic imaging is used to confirm the presence of a clinically suspected CR. The diagnostic accuracy of imaging is thought to be limited, because asymptomatic radiological abnormalities are commonly seen with advanced imaging studies [46]. This also holds true for plain X-ray studies that exhibit degenerative changes increasing with age unrelated to clinical signs and symptoms [1]. Most often MRI was used, although data concerning the sensitivity and specificity for the diagnosis of CR for MRI are sparse and questionable [46, 47]. A more recent study on the interobserver reliability of MRI evaluation in patients with CR has shown it to be substantial for nerve root compression, with or without previous clinical information [48]. In our study, only RCTs in a conservative treatment group were included, implying that we selected a group of patients often seen in primary health-care settings. We suppose that diagnostic imaging is much less often used in this setting, as the access to it is limited. This is supported by the North American spine surgeons’ clinical guidelines for the diagnosis and treatment of CR from degenerative disorders. They recommended that MRI was suggested only for the confirmation of correlative compressive lesions in cervical spine patients who failed a course of conservative therapy and who may be candidates for interventional or surgical treatment [32].

### Strengths and limitations

This is the first SR on definitions and selection criteria for CR. Even though CR is often mentioned as a separate entity in the assessment of patients with neck pain, most studies use their own criteria to sub-classify patients with CR, making comparisons difficult.

One of our limitations was that we only included published RCTs. Relevant unpublished trials were not included, thus potentially leading to publication bias. However, if they exist, these studies are likely to be small and to increase heterogeneity and therefore are unlikely to change our results [49].

For the purpose of this review, we have chosen to exclude studies that only included surgical interventions, as surgical studies may possibly recruit a different group of patients to justify the need for an invasive treatment.

We recommend to aim for consensus on a definition of CR and selection criteria among experts in this area, preferably with criteria that are quantifiable with validated clinical tests. Data from two recent RCTs support the clinical finding that patients usually report experiencing more arm pain than neck pain [19, 22]. We therefore suggest defining CR as: “Radiating pain in the arm with motor, reflex and/or sensory changes (such as paraesthesiae or numbness), provoked by neck posture(s) and/or movement(s)”. It is the researchers’ intent to conduct a Delphi study on this definition among different international researchers and practitioners. We would suggest the following selection criteria: pain radiating into the arm and motor, reflex, and/or sensory changes in the upper limb such as paraesthesia or numbness.

Future research should aim at the validity of selection criteria.

## Conclusion

This systematic review found no uniformity in definitions of CR. The criteria used to select patients with CR in interventional studies vary widely between different studies. We found consensus on only one criterion, which is neck and/or arm pain to select patients with CR for RCTs using conservative therapy as an intervention. We found no contradictory criteria. The selection criteria and test method used are poorly described.

## Appendix

See Table 2

**Table 2:** Medline search history

Search	Query	Items found
#18	Search #16 AND #17	148
#17	Search (random[tiab] OR randomized controlled trial[pt] OR randomized controlled trial[TW] OR randomised controlled trial[pt] OR randomised controlled trial[TW] OR double-blind method[TW] OR single-blind method[TW] OR placebo[TW] OR clinical trial[TW] OR controlled clinical trial[TW])	812,251
#16	Search #13 AND #15	1,543
#15	Search #5 OR #14	200,718
#14	Search “Neck”[tiab] OR “neck pain”[tiab] OR “Neck injury”[tiab] OR “Neck injuries”[tiab] OR “cervical rib syndrome”[tiab] OR “cervical rib”[tiab] OR “cervical plexus”[tiab] OR “cervical vertebrae”[tiab] OR “spondylosis”[tiab] OR spondyloses[tiab] OR “spinal manipulations”[tiab] OR “spinal manipulation”[tiab] OR “brachial plexus neuropathies”[tiab] OR “brachial plexus neuropathy”[tiab] OR “torticollis”[tiab] OR “lordosis”[tiab] OR “brachial plexus”[tiab]	140,911
#13	Search #4 OR #11	5,033
#11	Search Radiculopathy[tiab] OR Radiculopathies[tiab] OR Cervical Radiculopathy[tiab] OR Cervical Radiculopathies[tiab] OR Cervical Radiculopathy[tiab] OR Cervical Radiculopathie[tiab] OR Nerve Root Disorder[tiab] OR Nerve Root Disorders[tiab] OR Radiculitis[tiab] OR Radiculitides[tiab] OR Nerve Root Inflammation[tiab] OR Nerve Root Inflammations[tiab] OR Nerve Root Avulsion[tiab] OR Nerve Root Avulsions[tiab] OR Nerve Root Compression[tiab] OR Nerve Root Compressions[tiab]	4,766
#12	Search #4 AND #11	272
#10	Search #9 AND #7	1,881
#9	Search Radiculopathy[TW] OR Radiculopathies[TW] OR Cervical Radiculopathy[TW] OR Cervical Radiculopathies[TW] OR Cervical Radiculopathy[TW] OR Cervical Radiculopathie[TW] OR Nerve Root Disorder[TW] OR Nerve Root Disorders[TW] OR Radiculitis[TW] OR Radiculitides[TW] OR Nerve Root Inflammation[TW] OR Nerve Root Inflammations[TW] OR Nerve Root Avulsion[TW] OR Nerve Root Avulsions[TW] OR Nerve Root Compression[TW] OR Nerve Root Compressions[TW]	6,372
#8	Search #4 AND #7	180
#7	Search #5 OR #6	220,205
#6	Search “Neck”[TW] OR “neck pain”[TW] OR “Neck injury”[TW] OR “Neck injuries”[TW] OR “cervical rib syndrome”[TW] OR “cervical rib”[TW] OR “cervical plexus”[TW] OR “cervical vertebrae”[TW] OR “spondylosis”[TW] OR spondyloses[TW] OR “spinal manipulations”[TW] OR “spinal manipulation”[TW] OR “brachial plexus neuropathies”[TW] OR “brachial plexus neuropathy”[TW] OR “torticollis”[TW] OR “lordosis”[TW] OR “brachial plexus”[TW]	196,297
#5	Search “Neck”[MH] OR “neck pain”[MH] OR “Neck injuries”[MH] OR “cervical rib syndrome”[MH] OR “cervical rib”[MH] OR “cervical plexus”[MH] OR “cervical vertebrae”[MH] OR “spondylosis”[MH] OR “manipulation, spinal”[MH] OR “brachial plexus neuropathies”[MH] OR “torticollis”[MH] OR “lordosis”[MH] OR “brachial plexus”[MH]	87,560
#4	Search #1 NOT #2	539
#3	Search (“Radiculopathy/drug therapy”[Mesh] OR “Radiculopathy/prevention and control”[Mesh] OR “Radiculopathy/radiotherapy”[Mesh] OR “Radiculopathy/rehabilitation”[Mesh] OR “Radiculopathy/surgery”[Mesh] OR “Radiculopathy/therapy”[Mesh])	1,489
#2	Search (“Radiculopathy/blood”[Mesh] OR “Radiculopathy/cerebrospinal fluid”[Mesh] OR “Radiculopathy/chemically induced”[Mesh] OR “Radiculopathy/classification”[Mesh] OR “Radiculopathy/complications”[Mesh] OR “Radiculopathy/congenital”[Mesh] OR “Radiculopathy/diagnosis”[Mesh] OR “Radiculopathy/economics”[Mesh] OR “Radiculopathy/embryology”[Mesh] OR “Radiculopathy/enzymology”[Mesh] OR “Radiculopathy/epidemiology”[Mesh] OR “Radiculopathy/etiology”[Mesh] OR “Radiculopathy/genetics”[Mesh] OR “Radiculopathy/history”[Mesh] OR “Radiculopathy/immunology”[Mesh] OR “Radiculopathy/microbiology”[Mesh] OR “Radiculopathy/mortality”[Mesh] OR “Radiculopathy/nursing”[Mesh] OR “Radiculopathy/parasitology”[Mesh] OR “Radiculopathy/pathology”[Mesh] OR “Radiculopathy/physiopathology”[Mesh] OR “Radiculopathy/psychology”[Mesh] OR “Radiculopathy/radiography”[Mesh] OR “Radiculopathy/radionuclide imaging”[Mesh] OR “Radiculopathy/ultrasonography”[Mesh] OR “Radiculopathy/urine”[Mesh] OR “Radiculopathy/veterinary”[Mesh] OR “Radiculopathy/virology”[Mesh])	2,516
#1	Search “Radiculopathy”[Mesh]	3,055
